# Idiopathic Carpal Spasm (Trousseau’s Sign) After Intraoperative Arm Tourniquet Inflation in an Intubated Patient

**DOI:** 10.7759/cureus.7543

**Published:** 2020-04-04

**Authors:** Suzanne M Beecher, Stephanie M Bollard, Eamon S Beausang

**Affiliations:** 1 Plastic & Reconstructive Surgery, Cork University Hospital, Cork, IRL; 2 Plastic Surgery, Mater Misericordiae University Hospital, Dublin, IRL; 3 School of Medicine, University College Dublin, Dublin, IRL; 4 Plastic Surgery, St. James's Hospital, Dublin, IRL

**Keywords:** carpal spasm, tourniquet, trousseau’s sign

## Abstract

We present a case of a 38-year-old male who sustained a laceration from a knife to the volar aspect of his left index and middle fingers. He had clinical injury to his flexor digitorum profundus tendons to both digits. He underwent operative exploration and repair of the tendons under general anaesthetic. An arm tourniquet was inflated to allow for haemostasis in the operative field. A few minutes after inflation, the patient’s hand went into carpal spasm. The tourniquet was deflated and the spasm resolved. Intraoperative serum calcium and carbon dioxide levels were normal. The operation proceeded with the tourniquet deflated. Postoperatively serum calcium and magnesium levels were within normal limits, as was serum vitamin D and parathyroid hormone levels. It has been reported that carpal spasm can occur with tourniquet use in the anxious patient due to hyperventilation and resultant metabolic alkalosis. This however is the first reported case of carpal spasm in the setting of tourniquet use and normal serum electrolytes and respiratory parameters in an intubated patient.

## Introduction

Carpal spasm is classically a sign of hypocalcaemia. It can also be a sign associated with low serum magnesium, potassium or phosphate [1,2]. It can also occur in respiratory alkalosis due to a transient hypocalcaemia. It has been reported that carpal spasm can occur with tourniquet use under brachial blockade in the anxious patient due to hyperventilation and resultant respiratory alkalosis [3]. We however present the first case of carpal spasm with tourniquet use under general anaesthesia in the setting of a normal serum calcium and blood pH. 

## Case presentation

**Figure 1:**
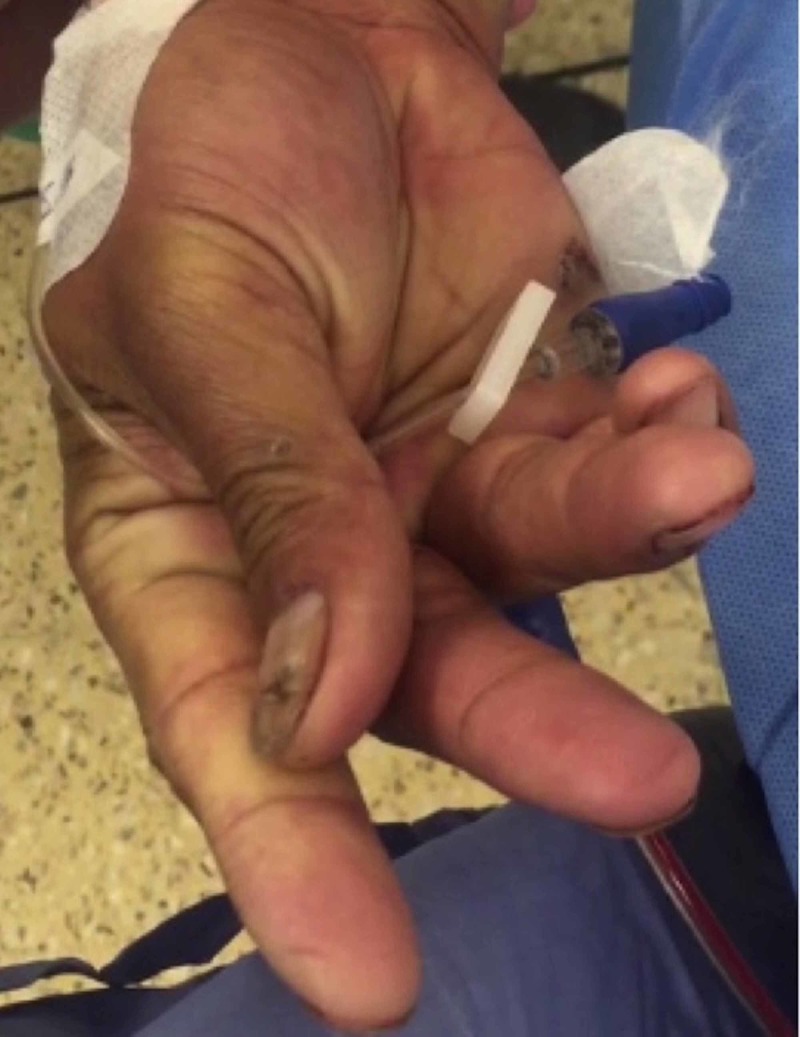
Carpal spasm of the right hand

A 38-year-old male was referred to our plastic surgery trauma clinic with a laceration to his left index and middle fingers. He was allegedly assaulted with a knife and sustained lacerations to the volar aspect of the middle phalanges. On examination, he had absent movement into flexor digitorum profundus tendons to both digits. Sensation was also reduced on the radial border of his middle finger. Both digits were perfused. He was admitted and underwent surgical exploration under general anaesthetic the next day. After skin preparation and draping, the upper arm tourniquet was inflated to 250 mmHg. A few minutes after inflation, the patient’s hand went into carpal spasm, with flexion at the wrist and metacarpophalangeal joints and extension of the proximal and distal interphalangeal joints (Figure 1).

The fingers were held in adduction. The tourniquet was immediately deflated, and the spasm resolved. The arm tourniquet was again inflated, and the hand again went into spasm. Intraoperative blood gas measurements were taken at this time and were normal, including ionized calcium levels and carbon dioxide levels. There were no abnormal ECG changes. The tourniquet was then deflated, and the operation proceeded without tourniquet control. The patient required repair of the flexor digitorum profundus tendons to his left index and middle fingers in zone 2. At the end of the operation, prior to extubation, an arm tourniquet was inflated on the contralateral arm. Again, after a few minutes, his hand went into spasm (Video 1).

**Video 1:** Carpal spasm of the right hand

Postoperatively serum calcium and magnesium levels were within normal limits, as was serum vitamin D and parathyroid hormone levels. The spasm did not occur with conventional use of a sphygmomanometer for blood pressure measurements. The patient did not report a history of carpal spasm. The patient made an unremarkable recovery and was discharged day 1 postoperatively.

## Discussion

Trousseau’s sign of latent tetany was first coined by the French physician Armand Trousseau in 1861. He described tetany of the hand as follows: “the thumb is forcibly and violently adducted; the fingers are pressed closely together, and semi-flexed over the thumb in consequence of the flexion of the metacarpophalangeal articulation; and the palm, of the hand being made hollow by the approximation of its outer and inner margins, the hand assumes a conical shape” [4]. The sign is also known as main d'accoucheur, as Trousseau reported that it resembles the position of an obstetrician’s hand in delivering a baby. This sign is classically associated with hypocalcaemia [5]. It can also be seen with low serum magnesium, phosphate and potassium [1,2]. The sign can be elicited in patients with low serum calcium by inflated an arm tourniquet for a few minutes. The low serum calcium and subsequent neuromuscular excitability causes spasm of the muscles of the hand and forearm. Another sign of low serum calcium includes Chovstek’s sign, which can be elicited by tapping over the facial nerve, leading to twitching of the facial muscles. Trousseau’s sign however is more sensitive and specific for hypocalcaemia [6].

In our study, we noted that both hands and forearms went into spasm after inflation of an upper arm tourniquet. Our patient’s serum calcium however was normal. There is one case report in the literature of this occurring in the setting of normal serum calcium levels [3]. It was proposed that it occurred due to hyperventilation in the anxious patient as the case was performed under brachial blockade. Hyperventilation leads to a respiratory alkalosis and transient hypocalcaemia, which can result in carpal spasm. An anxiolytic can successfully result in relaxation of the patient and cessation of carpal spasm in such cases [3]. In our case, however, the patient was intubated, with normal respiratory parameters, as well as normal calcium and electrolyte levels and no evidence of alkalosis on blood gas analysis. This is the first such case reported in the intubated patient.

It is possible that some anaesthetic agents, such as sevoflurane, can induce carpal spasm [7]. The aetiology of this is that the anaesthetic metabolite can possibly influence calcium homeostasis. This was however was not present in our case, as the patient had a normal serum calcium level measurement during the anaesthetic. 

The operation in this case proceeded without the aid of tourniquet control. It should be noted, however, that if tourniquet control was necessary, neuromuscular blockade might have been employed to relieve the spasm. Not only can neuromuscular blockade relieve muscular reactivity and spasm, it may also have a protective effect on muscle, with reduced muscle ischaemia in the setting of tourniquet use [8].

## Conclusions

This is the first reported case of carpal spasm in the setting of tourniquet use and normal serum electrolytes and respiratory parameters in the intubated patient. We are not sure of the aetiology of the carpal spasm as all investigations were normal. Carpal spasm can obscure the surgical field when performing surgery on the hand. The tourniquet must be deflated in order to proceed with surgery.
